# Comparison of SNP Genotypes Related to Proliferative Vitreoretinopathy (PVR) across Slovenian and European Subpopulations

**DOI:** 10.1155/2018/8761625

**Published:** 2018-05-15

**Authors:** Xhevat Lumi, Mateja M. Jelen, Daša Jevšinek Skok, Emanuela Boštjančič, Metka Ravnik-Glavač, Marko Hawlina, Damjan Glavač

**Affiliations:** ^1^Eye Hospital, University Medical Centre Ljubljana, Ljubljana, Slovenia; ^2^Department of Molecular Genetics, Institute of Pathology, Faculty of Medicine, University of Ljubljana, Ljubljana, Slovenia

## Abstract

The present study investigated the distribution of genotypes within single nucleotide polymorphisms (SNPs) in genes, related to PVR pathogenesis across European subpopulations. Genotype distributions of 42 SNPs among 96 Slovenian healthy controls were investigated and compared to genotype frequencies in 503 European individuals (Ensembl database) and their subpopulations. Furthermore, a case-control status was simulated to evaluate effects of allele frequency changes on statistically significant results in gene-association studies investigating functional polymorphisms. In addition, 96 healthy controls were investigated within 4 SNPs: rs17561 (*IL1A*), rs2069763 (*IL2*), rs2229094 (*LTA*), and rs1800629 (*TNF*) in comparison to PVR patients. Significant differences (*P* < 0.05) in distribution of genotypes among 96 Slovenian participants and a European population were found in 10 SNPs: rs3024498 (*IL10*), rs315952 (*IL1RN*), rs2256965 (*LST1*), rs2256974 (*LST1*), rs909253 (*LTA*), rs2857602 (*LTA*), rs3138045 (*NFKB1A*), rs3138056 (*NFKB1A*), rs7656613 (*PDGFRA*), and rs1891467 (*TGFB2*), which additionally showed significant differences in genotype distribution among European subpopulations. This analysis also showed statistically significant differences in genotype distributions between healthy controls and PVR patients in rs17561 of the *IL1A* gene (OR, 3.00; 95% CI, 0.77–11.75; *P* = 0.036) and in rs1800629 of the *TNF* gene (OR, 0.48; 95% CI, 0.27–0.87; *P* = 0.014). Furthermore, we have shown that a small change (0.02) in minor allele frequency (MAF) significantly affects the statistical *p* value in case-control studies. In conclusion, the study showed differences in genotype distributions in healthy populations across different European countries. Differences in distribution of genotypes may have had influenced failed replication results in previous PVR-related SNP-association studies.

## 1. Introduction

The impact of genome-wide association studies and genetic-association studies has become enormous in the past ten years, providing researchers with extensive data repositories [1]. As genetic factors affect susceptibility to certain diseases, identifying the relevant genes and/or their polymorphisms contribute greatly to the development of novel prevention programs and treatments of disease. Numerous evaluations of genetic association have also led to the remarkable potential for the discovery of novel genetic biomarkers. However, the execution of such analysis in many cases is cumbersome with considerable statistical and computational challenges and also requires reproducibility [2]. The potential for the discovery of false positive findings when results are not properly corrected is high and represents the most conspicuous problem in gene-disease-association studies [3–5].

Proliferative vitreoretinopathy (PVR) represents the growth and contraction of cellular membranes on both retinal surfaces and within the vitreous cavity in patients with rhegmatogenous retinal detachment (RRD) [6–8]. It is the major complication following retinal detachment surgery and a leading cause of failure in the management of RRD [6, 9]. It is estimated to occur in 5–10% of patients with RRD [6]. Technological advances in high-throughput screening have been introduced in gene-association studies, including PVR. These studies revealed numerous inflammatory molecules to be implicated in the PVR development, such as growth factors (PDGF, HGF, VEGF, and EGF), transforming growth factors (TGFA, TGFB), molecules from the SMAD family and interleukins (IL1, IL6, IL8, and IL10), tumor necrosis factors (TNF), and tumor suppressor protein (p53) [10–15]. Studies, published in the past ten years, by the “Retina 4 Project” consortium, have demonstrated that specific single nucleotide polymorphisms (SNPs), located in genes involved in PVR pathways, may represent potential predictive factors for the PVR development [10, 11, 14, 16–20]. Among 200 studied SNPs in more than 30 candidate genes, the “Retina 4 Project” identified 8 SNPs in 7 genes, encoding CCL2, FGF2, IL1RN, LTA, NFKBIA, SMAD7, and TGFB2, as significant individual predictors for PVR [11] and demonstrated associations between PVR and SNPs in *BAX*, *p53*, *PIK3CG*, *MDM2*, *SMAD7*, and *TNFB2* in the TNF locus [10, 16–19]. A more recent genetic-association study, on a Slovenian PVR patient population, demonstrated significant differences in genotype distributions between RRD patients with and without PVR in SNPs within *IL6*, in the vicinity of *IL10*, and the *TGFB1* gene. Interestingly, several associations between SNP genotypes and the PVR phenotype could not be replicated throughout a series of “Retina 4 Project” studies and by a recent study on a Slovenian population. To establish the credibility of an association between a SNP and disease, a replication of SNP effect among different study populations is essential. It is possible that fluctuations between genotype frequencies across studied countries reflect the difference in population ancestry, which could influence the variability in allele frequencies even in unrelated conditions of interest [21]. As success of replication of a genetic-association study depends on many factors, including enrollment of independent population datasets, information on the effect of different allele frequencies in genetic-association studies remains scarce.

The present study is a part of an ongoing gene-association study in Slovenian RRD patients who developed PVR after vitrectomy. In order to expand the current perspective on differences between PVR patients and healthy controls and SNP effects in patients with different geographical background, we further investigated our previously established in-house genomic databases. Firstly, we aimed to assess basic differences in SNP genotype distributions among European subpopulations. For this purpose, we compared distributions of 42 SNP genotypes between a Slovenian healthy population and European subpopulations and among European subpopulations. Additionally, this study designed a simulation of a case-control gene-association study in order to demonstrate that even a minor allele frequency (MAF) change could result in a considerable increase in the power to replicate the previously established SNP effect. In the second part of the study, we examined differences in distribution of SNP genotypes between Slovenian healthy controls and PVR patients.

## 2. Methods

### 2.1. Study Population

The genetic-association study conducted on 191 Slovenian patients with primary RRD, who underwent vitrectomy at the Eye Hospital, University Medical Centre Ljubljana, Slovenia. In the study we recruited 153 patients who developed PVR grade C1 or higher within 3 months after the surgery. We also enrolled 96 healthy controls without retinal detachment. The study was approved by the National Medical Ethics Committee of the Republic of Slovenia and followed the tenets of the Helsinki Declaration. All patients provided written informed consent.

Ninety-six healthy Slovenian blood donors (52 men and 44 women), aged between 20 and 55 years, originating from 11 geographic areas, representative for the country of Slovenia, were statistically analyzed in 4 SNPs.

### 2.2. Blood Collection and DNA Extraction

Six milliliters of peripheral blood were collected from each participant and stored until DNA extraction at −20°C. DNA was extracted using QIAamp DNA Blood Midi Kit (100) (QIAGEN, Hilden, Germany), according to the manufacturer's instructions. Extracted DNA was stored until used for amplification at −20°C.

### 2.3. Genotype Distribution in Slovenian and European Populations

Genotype distributions of 42 SNPs of 96 Slovenian healthy controls, genotyped using HumanOmniExpress-14 platform (Illumina, San Diego, CA, USA), were compared across 503 European residents, using data on specific SNP genotypes obtained from the Ensembl database (release 83) (Supplemental Table 1). In case a difference among SNP genotype distribution was observed among Slovenian and European populations, the differences between the populations were further examined, as follows: the frequencies of genotypes were subsequently compared between the Slovenian and three European subpopulations, namely, 99 Utah residents with northern and western European ancestry (CEU), 91 residents from Britain in England and Scotland (GBR), and 107 Iberian residents from Spain (IBS).

### 2.4. Evaluation of Genetic Effects in a Simulated Population Dataset

We hypothesized that some statistically significant differences in SNP genotype distributions could not be replicated due to even small changes in allele frequencies. To test this hypothesis, we designed a simulated case-control status. The genotype frequency of the original case dataset was AA = 1, AG = 39, and GG = 73, and the minor allele frequency (MAF) was 0.18 (*P* = 0.13). The control dataset remained unchanged. Genotypes were added one by one in each homozygote or heterozygote category to evaluate effects of minor allele frequency (MAF) changes on statistically significant results (Table 1).

### 2.5. Genotyping of 96 Healthy Participants

Ninety-six Slovenian healthy controls were genotyped for 713,014 markers, using HumanOmniExpress-14 platform (Illumina, San Diego, CA, USA). Genotypes were assigned according to the standard Illumina protocol in GenomeStudio Software, version V2011.1. Only individuals with a genotyping success rate of >95% were considered as positive for a respective genotype. The HumanOmniExpress-14 platform included only 4 SNPs, investigated previously in RRD and PVR patients. Therefore, our subsequent analysis included comparison of 96 control PVR patients for 4 SNPs: rs17561 (*IL1A*), rs2069763 (*IL2*), rs229094 (*LTA*) and rs1800629 (*TNF*).

### 2.6. TaqMan Genotyping of PVR Patients

Genotypes of 4 SNPs, located within or in the vicinity of the 4 genes rs17561 (*IL1A*), rs2069763 (*IL2*), rs2229094 (*LTA*), and rs1800629 (*TNF*), were determined using TaqMan assay (Applied Biosystems, Foster City, CA, USA) according to the manufacturer's instructions.

### 2.7. Statistical Analysis

**Figure 1:**
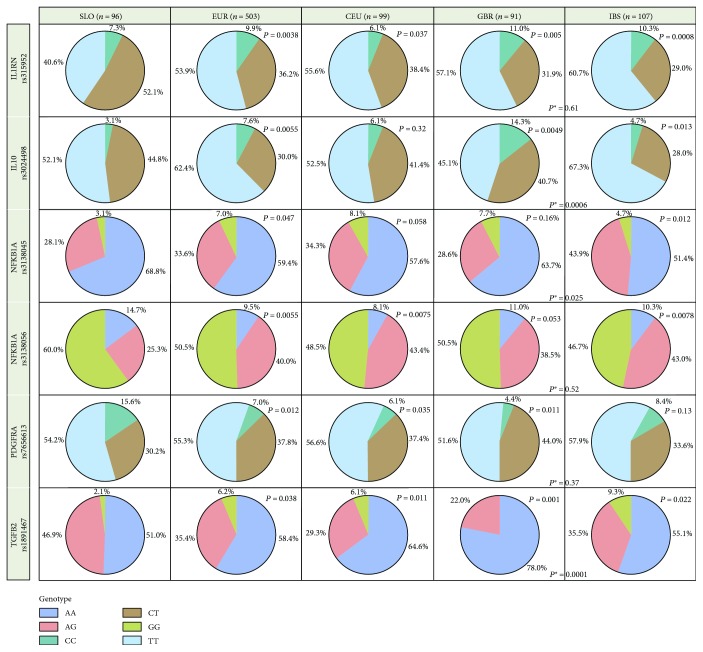
The genotype frequencies for 6 SNPs across European subpopulations. *P* value means difference in genotype distribution between Slovenian population (SLO) and other populations (EUR, CEU, GBR and IBS). *P* ^∗^ value means difference in genotype distribution between Great Britain population (GBR) and Iberian population (IBS) only.

**Figure 2:**
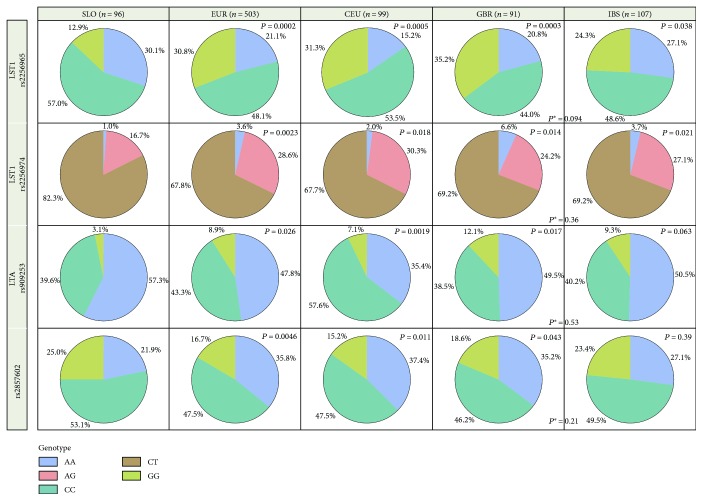
The genotype frequencies for 4 SNPs across European subpopulations. *P* value means difference in genotype distribution between Slovenian population (SLO) and other populations (EUR, CEU, GBR and IBS). *P* ^∗^ value means difference in genotype distribution between Great Britain population (GBR) and Iberian population (IBS) only.

Differences in genotype distributions among Slovenian healthy controls and European subpopulations were evaluated with the chi-square test, calculated using SAS software version 9.2 (JMP®, SAS Institute Inc., 2010, Cary, North Carolina, USA) and presented as pie charts (Figures 1 and 2).

To assess the differences in SNP genotype distribution among healthy population and PVR patients, odds ratios (ORs) with 95% confidence intervals (95% CIs) were calculated in SNPStats software [22], using the unconditional logistic regression. For inheritance model identification, the Akaike information criteria (AIC) were used, according to the authors' instructions. An *α* value was set to 0.05 in all calculations.

## 3. Results

The genotype distribution comparison of 42 SNPs among 96 Slovenian healthy controls (SLO) and a European population (data for 503 individuals were obtained from the Ensembl database) revealed significant differences (*P* < 0.05) in distribution of genotypes in 10 SNPs: rs3024498 (*IL10*), rs315952 (*IL1RN*), rs2256965 (*LST1*), rs2256974 (*LST1*), rs909253 (*LTA*), rs2857602 (*LTA*), rs3138045 (*NFKB1A*), rs3138056 (*NFKB1A*), rs7656613 (*PDGFRA*), and rs1891467 (*TGFB2*) (see Figures 1 and 2).

The frequencies of genotypes rs315952 (*IL1RN*), rs2256965 (*LST1*), and rs2256974 (*LST1*) varied significantly between SLO and CEU, SLO and GBR, and SLO and IBS, while the differences between GBR and IBS were not observed. Similar differences were observed for SNP rs1891467 (*TGFB2*), where we noticed the differences between all comparisons with the Slovenian population, as well as between GBR and IBS. Differences in the frequency of genotypes for the SNP rs3024498 (*IL10*) were observed between SLO and GRB, SLO and IBS, and between GBR and IBS. For the SNP rs3138056 (*NFKB1A*), differences between SLO and CEU and between SLO and IBS were observed. The differences in the frequencies of genotypes for the SNP rs3138045 (*NFKB1A*) were observed between SLO and IBS and GBR and IBS, while the frequencies of genotypes rs7656613 (*PDGFRA*), rs909253 (*LTA*), and rs2857602 (*LTA*) differ between populations of SLO and CEU and SLO and GBR.

Simulation of the potential population dataset of SNP genotypes revealed that adding six heterozygotes (AG) to the original case dataset showed a statistically significant difference between the two populations (*P* = 0.047) (Table 1). Similarly, statistically significant differences were shown when one homozygote (AA) and four heterozygotes (AG), or two homozygotes (AA) and two heterozygotes (AG), or three homozygotes (AA), were added to the original case dataset. Despite the fact that MAF increased from 0.18 to 0.20 in all described cases of events, the small change (0.02) in MAF showed an important decrease of the *P* value below 0.05.

In addition, the analysis showed two statistically significant differences in genotype distributions between 96 healthy controls and PVR patients (Table 2). In *IL1A* (rs17561), a statistically significant difference in distribution of genotypes was found between PVR patients and healthy controls (OR, 3.00; 95% CI, 0.77 to 11.75; *P* = 0.036). A significantly different distribution of genotypes was found also in rs1800629 of the *TNF* gene (OR, 0.48; 95% CI, 0.27 to 0.87; *P* = 0.014).

## 4. Discussion

Numerous inflammatory mediators, growth factors, and cytokines have been implicated in PVR pathogenesis. Statistical results of several genetic-association studies within the “Retina 4 Project” have emphasized the possible potential of those inflammatory mediators as novel biomarkers in the diagnostics and treatment of PVR [7, 8, 20, 23]. Replication of statistical results in gene-association studies has become the golden standard for assessing the independent effect of SNP and/or its genomic location to a certain disease [3]. Unfortunately, reproducibility is frequently challenging to achieve due to genetic heterogeneity, inadequate population size, or variability in phenotype definitions, environmental interactions, inadequate statistical power, and age-dependent effects [1, 2, 24–26].

Previous gene-association studies investigating SNPs in PVR have demonstrated significant differences between PVR cases, RRD controls, and healthy controls and predicted several genetic associations for PVR development [10, 16–19]. Fundamental studies in PVR research were based on international investigation of SNP genotype associations and included patients from Spain, Portugal, UK, and Netherlands [18, 19]. However, these studies did not include the comparison of genotype distributions in healthy populations across European subpopulations. For this reason, it is possible that failed replications of SNP effects in studies that followed were a consequence of different genetic structures across studied populations *per se*.

The present study compared the distribution of 42 SNP genotypes between Slovenian and European healthy populations and revealed significant differences in 10 SNPs, suggesting a somewhat similar distribution of genotypes among residents of common European ancestry. Our results firstly suggest that genotype polymorphisms more frequently identified in individuals from one European country could probably share a similar genotype pattern in individuals from other European countries as well. Our observations also indicate that different allele frequencies across independent datasets indeed influence the final SNP effect, frequently leading to spurious results in replication studies. The mentioned bias has been confirmed in our study by manipulating a simulated case-control dataset, which revealed that already a small change of 0.02 in MAF indeed causes important differences in statistical significance in genetic-association studies. Similar results were obtained in a simulation study of two interacting SNPs by Greene et al. which showed that the power to replicate the statistically significant independent effect of one SNP can drop dramatically with a change in allele frequency of less than 0.1 at the second interacting polymorphism [3]. On the other hand, it has been proposed that population structure has so far caused less inaccurate associations in genetic-association studies than it was initially predicted. When systematic ethnicity matching and application of standard quality control measures are not provided by research executors, population effect can still represent a major bias in these studies [5]. In the second part of our study, we have found a statistically significant difference in distribution of genotypes between healthy controls and PVR patients in rs17561 within the *IL1A* gene and rs1800629 (*TNF*). Similar significance was observed in a previously published study by Sanabria Ruiz-Colmenares et al. for *TGFB1* (rs1800471), when no significant difference in genotype distribution was observed between patients with and without PVR; instead, a statistically significant difference was observed between PVR patients and healthy controls [14].

The impact of different distributions of genotypes in SNPs in TNF locus, which encodes also TNF-*α*, has been investigated in three subsequent studies by a Spanish group of the Retina 4 Project [10, 11, 16]. Various SNPs in *TNF* have been shown to be associated with increased risk of PVR development, including the rs1800629. However, we have found a statistically significant difference in distributions of genotypes between healthy controls and PVR patients. In conclusion, the ultimate goal in PVR research as well as in other human diseases is to detect genetic associations, which replicate in studies without a significant bias. Our study showed that differences in genotype distributions exist between healthy populations across different European countries and may have had influenced the failed replication results in PVR SNP-association studies. This study confirmed the importance of baseline screening of the healthy population before investigating patients originating from the same dataset. Considering the fact that genotype distributions in patients with PVR and RRD patients without PVR have been compared within a limited number of European countries (Netherlands, Portugal, Slovenia, Spain, and United Kingdom), and that results of different previous PVR studies failed to be replicated, it is crucial to conduct larger multicentric population-based study.

## Figures and Tables

**Table 1:** Simulation of genotype distribution in a potential population dataset.

Number of simulations	Genotype case dataset (*n*)			Number of genotypes added to the case dataset (*n*)			Allele frequency		MAF	*P* value
	AA	AG	GG	AA	AG	GG	A	G		
1	1	39	73	0	0	0	0.18	0.82	0.18	0.130
2	1	40	73	0	1	0	0.18	0.82	0.18	0.110
3	1	41	73	0	2	0	0.19	0.81	0.19	0.092
4	1	42	73	0	3	0	0.19	0.81	0.19	0.078
5	1	43	73	0	4	0	0.19	0.81	0.19	0.066
6	1	44	73	0	5	0	0.19	0.81	0.19	0.056
7	1	45	73	0	6	0	0.20	0.80	0.20	0.047
8	2	39	73	1	0	0	0.19	0.81	0.19	0.091
9	2	40	73	1	1	0	0.19	0.81	0.19	0.077
10	2	41	73	1	2	0	0.19	0.81	0.19	0.065
11	2	42	73	1	3	0	0.20	0.80	0.20	0.055
12	2	43	73	1	4	0	0.20	0.80	0.20	0.047
13	3	39	73	2	0	0	0.20	0.80	0.20	0.065
14	3	40	73	2	1	0	0.20	0.80	0.20	0.055
15	3	41	73	2	2	0	0.20	0.80	0.20	0.046
16	4	39	73	3	0	0	0.20	0.80	0.20	0.041

Note: original case dataset is shown in the second column. The control dataset is not shown. Added genotypes to the original dataset are represented in the third column. Genotypes were added one by one in each homozygote or heterozygote category. Allele frequency, MAF, and *P* values changed according to the performed simulation.

**Table 2:** Genotype distributions of 4 SNPs in Slovenian patients with PVR and 96 healthy controls. Inheritance models and odds ratios (ORs) were determined.

Gene	SNP	Genotype	Genotype frequency in healthy controls (%)	Genotype frequency in cases (%)	Inheritance model^∗^	OR (95% CI)	*P* value
IL1A	rs17561	CC	49 (51)	49 (43.39)	Codominant (CC-CA/AA)	3.00 (0.77–11.75)	**0.036**
	C/A	CA	38 (40)	59 (52.29)			
		AA	9 (9)	3 (2.7)			
		ND	0 (0)	2 (1.8)			
Total number of participants			96 (100)	113 (100)			

IL2	rs2069763	CC	39 (41)	52 (46.0)	Recessive^∗∗^ (CC/CA-AA)	1.51 (0.71–3.18)	0.28
	C/A	CA	39 (41)	46 (40.7)			
		AA	18 (19)	15 (13.3)			
Total number of participants			96 (100)	113 (100)			

LTA	rs2229094	CC	8 (8.3)	15 (9.8)	Additive	1.15 (0.78–1.70)	0.49
	T/C	TC	33 (34.4)	55 (36.0)			
		TT	55 (57.3)	79 (51.6)			
		ND	0 (0)	4 (2.6)			
Total number of participants			96 (100)	153 (100)			

TNF	rs1800629	GG	74 (77)	96 (62.7)	Overdominant (GG-AA/AG)	0.48	**0.014**
	G/A	AG	20 (21)	54 (35.3)		0.27–0.87	
		AA	2 (2)	3 (2.0)			
Total number of participants			96 (100)	153 (100)			

Abbreviations: OR: odds ratio; 95% CI: 95% confidence interval; ND: patients, in which genotype could not be identified. ^∗^Inheritance models: additive: each copy of the rare variant modifies the risk; dominant: a single copy of the frequent variant is enough to modify the risk; recessive: two copies of the variant allele are necessary to change the risk; overdominant: heterozygosity modifies the risk. ^∗∗^In case of IL2, the inheritance model could be also additive (OR, 1.23; 95% CI, 0.84–1.80; *P* = 0.28).

## Supplementary Materials

Supplementary Materials Supplemental Table 1: genotype distributions of 42 SNPs in a Slovenian population (*n* = 96) versus a European population (*n* = 503).
