# Supplementary material for: Comparison of SNP Genotypes Related to Proliferative Vitreoretinopathy (PVR) across Slovenian and European Subpopulations
Source: J Ophthalmol. 2018 May 15;2018:8761625. doi: 10.1155/2018/8761625 (PMC5976970; doi:10.1155/2018/8761625)
Supplement: Supplementary Materials — Supplemental Table 1: genotype distributions of 42 SNPs in a Slovenian population (n = 96) versus a European population (n = 503). [file 8761625.f1.docx]

**Supplemental table 1:** Genotype distributions of 42 SNPs in Slovenian population (n=96) vs. European population (n=503).

| Gene | SNP | Genotype | SLO | EUR | Inheritance model | OR (95% Cl) | P value |
| --- | --- | --- | --- | --- | --- | --- | --- |
| ABCB1 | rs1128503 | GG | 29 (30.2) | 168 (33.4) | Additive | 1.12 (0.82-1.54) | 0.48 |
|  | A/G | AG | 49 (51.0) | 252 (50.1) |  |  |  |
|  |  | AA | 18 (18.8) | 83 (16.5) |  |  |  |
| ABCB1 | rs2235035 | CC | 39 (40.6) | 228 (45.3) | Recessive (CC-CT/TT) | 1.62 (0.85-3.07) | 0.15 |
|  | C/T | CT | 43 (44.8) | 227 (45.1) |  |  |  |
|  |  | TT | 14 (14.6) | 48 (9.6) |  |  |  |
| ABCB1** | rs2032582 | TT | 21 (21.9) | 80 (16.5) | Additive | 1.26 (0.92-1.74) | 0.15 |
|  | T/C | TC | 49 (51.0) | 246 (50.7) |  |  |  |
|  |  | CC | 26 (27.1) | 159 (32.8) |  |  |  |
| ABCB1 | rs1045642 | TT | 22 (22.9) | 134 (26.6) | Dominant (TT/CT-CC) | 1.22 (0.73-2.05) | 0.44 |
|  | C/T | CT | 51 (53.1) | 253 (50.3) |  |  |  |
|  |  | CC | 23 (24.0) | 116 (23.1) |  |  |  |
| CKAP2L | rs2048874 | TT | 1 (1.0) | 5 (1.0) | Overdominant (CC/TT-CT) | 1.57 (0.97-2.53) | 0.071 |
|  | C/T | CT | 30 (31.3) | 113 (22.5) |  |  |  |
|  |  | CC | 65 (67.7) | 385 (76.5) |  |  |  |
| CXCL8 | rs2227543 | TT | 20 (20.8) | 80 (15.9) | Recessive (CC-CT/TT) | 1.39 (0.80-2.41) | 0.25 |
|  | C/T | CT | 44 (45.8) | 235 (46.7) |  |  |  |
|  |  | CC | 32 (33.3) | 188 (37.4) |  |  |  |
| IL1A | rs17561 | GG | 49 (51) | 256 (50.9) | Recessive (GG-GT/TT) | 1.14 (0.53-2.42) | 0.74 |
|  | G/T | GT | 38 (39.6) | 205 (40.8) |  |  |  |
|  |  | TT | 9 (9.4) | 42 (8.3) |  |  |  |
| IL1A* | rs1800587 | TT | 9 (10.1) | 42 (8.3) | Additive | 1.11 (0.79-1.56) | 0.56 |
|  | C/T | CT | 37 (41.6) | 205 (40.8) |  |  |  |
|  |  | CC | 43 (48.3) | 256 (50.9) |  |  |  |
| IL1B | rs1143634 | TT | 3 (3.1) | 24 (4.7) | Additive | 0.84 (0.57-1.24) | 0.37 |
|  | C/T | CT | 36 (37.5) | 201 (40.0) |  |  |  |
|  |  | CC | 57 (59.4) | 278 (55.3) |  |  |  |
| IL1B | rs16944 | AA | 15 (15.6) | 62 (12.3) | Additive | 1.24 (0.91-1.71) | 0.18 |
|  | A/G | AG | 47 (49.0) | 228 (45.3) |  |  |  |
|  |  | GG | 34 (35.4) | 213 (42.4) |  |  |  |
| IL1B | rs3917368 | AA | 12 (12.5) | 63 (12.5) | Overdominant (GG-AA/AG) | 1.17 (0.76-1.82) | 0.48 |
|  | A/G | AG | 45 (46.9) | 216 (43.0) |  |  |  |
|  |  | GG | 39 (40.6) | 224 (44.5) |  |  |  |
| IL1RN | rs1688072 | AA | 65 (67.7) | 350 (69.6) | Dominant (AA/AG-GG) | 1.09 (0.68-1.74) | 0.72 |
|  | A/G | AG | 28 (29.2) | 139 (27.6) |  |  |  |
|  |  | GG | 3 (3.1) | 14 (2.8) |  |  |  |
| IL1RN | rs315952 | TT | 39 (40.6) | 271 (53.9) | Overdominant (TT-CC/CT) | 1.92 (1.23-2.98) | 0.0038 |
|  | C/T | CT | 50 (52.1) | 182 (36.2) |  |  |  |
|  |  | CC | 7 (7.3) | 50 (9.9) |  |  |  |
| IL2 | rs2069763 | GG | 39 (40.6) | 213 (42.4) | Recessive (GG-GT/TT) | 1.81 (1.01-3.23) | 0.055 |
|  | G/T | GT | 39 (40.6) | 233 (46.3) |  |  |  |
|  |  | TT | 18. (18.8) | 57 (11.3) |  |  |  |
| IL6 | rs1474347 | TT | 16 (16.7) | 181 (36.0) | Overdominant (TT-GG/TG) | 1.4 (0.91-2.17) | 0.13 |
|  | G/T | GT | 52 (54.2) | 230 (45.7) |  |  |  |
|  |  | GG | 28 (29.2) | 92 (18.3) |  |  |  |
| IL6 | rs2056576 | TT | 7 (7.3) | 49 (9.8) | Recessive (CC-CT/TT) | 0.73 (0.32-1.66) | 0.44 |
|  | C/T | CT | 45 (46.9) | 230 (45.7) |  |  |  |
|  |  | CC | 44 (45.8) | 224 (44.5) |  |  |  |
| IL10 | rs1800871 | AA | 6 (6.3) | 29 (5.8) | Overdominant (GG-AA/AG) | 0.79 (0.5-1.27) | 0.33 |
|  | A/G | AG | 30 (31.3) | 183 (36.3) |  |  |  |
|  |  | GG | 60 (62.5) | 291 (57.9) |  |  |  |
| IL10 | rs3024498 | TT | 50 (52.1) | 314 (62.4) | Overdominant (TT-CC/TC) | 1.89 (1.21-2.95) | 0.0055 |
|  | T/C | TC | 43 (44.8) | 151 (30.0) |  |  |  |
|  |  | CC | 3 (3.1) | 38 (7.6) |  |  |  |
| LST1*** | rs2256965 | GG | 12 (12.9) | 155 (30.8) | Dominant (GG/GA-AA) | 0.33 (0.18-0.63) | 0.0002 |
|  | G/A | GA | 53 (57.0) | 242 (48.1) |  |  |  |
|  |  | AA | 28 (30.1) | 106 (21.1) |  |  |  |
| LST1 | rs2256974 | GG | 1 (1.0) | 18 (3.6) | Additive | 2.08 (1.25-3.45) | 0.0023 |
|  | G/A | GA | 16 (16.7) | 144 (28.6) |  |  |  |
|  |  | AA | 79 (82.3) | 341 (67.8) |  |  |  |
| LTA | rs909253 | AA | 55 (57.3) | 240 (47.7) | Additive | 1.5 (1.04-2.17) | 0.026 |
|  | A/G | AG | 38 (39.6) | 218 (43.3) |  |  |  |
|  |  | GG | 3 (3.1) | 45 (9.0) |  |  |  |
| LTA | rs2857602 | AA | 21 (21.9) | 180 (35.8) | Additive | 0.64 (0.47-0.87) | 0.0046 |
|  | A/G | AG | 51 (53.1) | 239 (47.5) |  |  |  |
|  |  | GG | 24 (25.0) | 84 (16.7) |  |  |  |
| MIF | rs1007888 | TT | 26 (27.1) | 169 (33.6) | Overdominant (TT-CC/CT) | 1.36 (0.88-2.1) | 0.17 |
|  | C/T | CT | 51 (53.1) | 229 (45.5) |  |  |  |
|  |  | CC | 19 (19.8) | 105 (20.9) |  |  |  |
| MMP2 | rs243864 | TT | 62 (64.6) | 273 (54.3) | Overdominant (TT-GG/GT) | 0.63 (0.39-1.02) | 0.052 |
|  | G/T | GT | 27 (28.1) | 193 (38.4) |  |  |  |
|  |  | GG | 7 (7.3) | 37 (7.3) |  |  |  |
| MMP2 | rs243845 | GG | 37 (38.5) | 199 (39.6) | Recessive (GG-GA/AA) | 1.37 (0.78-2.38) | 0.28 |
|  | G/A | GA | 40 (41.7) | 227 (45.1) |  |  |  |
|  |  | AA | 19 (19.8) | 77 (15.3) |  |  |  |
| MMP2 | rs243866 | AA | 6 (6.3) | 33 (6.6) | Overdominant (GG-AA/AG) | 0.64 (0.4-1.04) | 0.065 |
|  | A/G | AG | 28 (29.2) | 196 (39.0) |  |  |  |
|  |  | GG | 62 (64.6) | 274 (54.4) |  |  |  |
| MMP2 | rs7201 | AA | 35 (36.5) | 151 (30.0) | Additive | 0.81 (0.59-1.11) | 0.18 |
|  | A/C | AC | 45 (46.9) | 248 (49.3) |  |  |  |
|  |  | CC | 16 (16.7) | 104 (20.7) |  |  |  |
| MMP9 | rs2250889 | CC | 86 (89.6) | 459 (91.3) | Recessive (CC-CG/GG) | 1.28 (0.62-2.64) | 0.4 |
|  | C/G | CG | 10 (10.4) | 42 (8.3) |  |  |  |
|  |  | GG | 0 (0) | 2 (0.4) |  |  |  |
| MMP9 | rs17577 | AA | 6 (6.3) | 13 (2.6) | Recessive (GG-AG/AA) | 2.51 (0.93-6.78) | 0.087 |
|  | A/G | AG | 22 (22.9) | 150 (29.8) |  |  |  |
|  |  | GG | 68 (70.8) | 340 (67.6) |  |  |  |
| NFKB1 | rs997476 | TT | 1 (1.0) | 3 (0.6) | Recessive (CC-AC/AA) | 1.75 (0.18-17.05) | 0.64 |
|  | G/T | GT | 9 (9.4) | 54 (10.7) |  |  |  |
|  |  | GG | 86 (89.6) | 446 (88.7) |  |  |  |
| NFKB1A | rs3138045 | AA | 66 (68.8) | 299 (59.4) | Additive | 0.68 (0.46-1.01) | 0.047 |
|  | A/G | AG | 27 (28.1) | 169 (33.6) |  |  |  |
|  |  | GG | 3 (3.1) | 35 (7.0) |  |  |  |
| NFKB1A | rs3138056 | TT | 14 (14.6) | 48 (9.5) | Overdoinant (CC-TT/CT) | 0.51 (0.31-0.82) | 0.0055 |
|  | C/T | CT | 24 (25.0) | 201 (40) |  |  |  |
|  |  | CC | 58 (60.4) | 254 (50.5) |  |  |  |
| NFKB1B | rs2241705 | AA | 54 (56.3) | 267 (53.1) | Overdominant (AA-CC/AC) | 0.77 (0.49-1.23) | 0.27 |
|  | A/C | AC | 31 (32.3) | 192 (38.2) |  |  |  |
|  |  | CC | 11 (11.5) | 44 (8.7) |  |  |  |
| p53 | rs1042522 | GG | 10 (10.4) | 37 (7.4) | Recessive (GG-CG/CC) | 1.46 (0.70-3.06) | 0.32 |
|  | C/G | CG | 36 (37.5) | 213 (42.3) |  |  |  |
|  |  | CC | 50 (52.1) | 253 (50.3) |  |  |  |
| PDGFRA | rs7656613 | CC | 15 (15.6) | 35 (7.0) | Recessive (TT-CT/CC) | 2.48 (1.29-4.74) | 0.012 |
|  | C/T | CT | 29 (30.2) | 190 (37.8) |  |  |  |
|  |  | TT | 52 (54.2) | 278 (55.2) |  |  |  |
| PIK3CG | rs4460309 | CC | 55 (57.3) | 271 (53.9) | Overdominant (CC-TT/CT) | 0.86 (0.54-1.35) | 0.51 |
|  | C/T | CT | 34 (35.4) | 196 (38.9) |  |  |  |
|  |  | TT | 7 (7.3) | 36 (7.2) |  |  |  |
| SIRT2 | rs10410544 | TT | 17 (17.7) | 89 (17.7) | Dominant (CC/CT-TT) | 0.85 (0.54-1.34) | 0.49 |
|  | C/T | CT | 44 (45.8) | 249 (49.5) |  |  |  |
|  |  | CC | 35 (36.5) | 165 (32.8) |  |  |  |
| SIRT2 | rs11879872 | TT | 56 (58.3) | 290 (57.6) | Recessive (TT-CT/CC) | 1.71 (0.81-3.61) | 0.18 |
|  | C/T | CT | 30 (31.3) | 181 (36.0) |  |  |  |
|  |  | CC | 10 (10.4) | 32 (6.4) |  |  |  |
| TGFB2 | rs1891467 | AA | 49 (51.0) | 294 (58.4) | Codominant (AA/AG/GG) | 1.52 (0.97-2.37) | 0.0038 |
|  | A/G | AG | 45 (46.9) | 178 (35.4) |  |  |  |
|  |  | GG | 2 (2.1) | 31 (6.2) |  |  |  |
| TNF | rs1800629 | GG | 74 (77.1) | 374 (74.4) | Overdominant (GG-AA/AG) | 0.81 (0.48-1.39) | 0.44 |
|  | A/G | AG | 20 (20.8) | 123 (24.4) |  |  |  |
|  |  | AA | 2 (2.1) | 6 (1.2) |  |  |  |

Abbreviations: *SLO; n=89, **EUR; n=485, ***SLO; n=93
